# Molecular-scale thermoelectricity: as simple as ‘ABC’†

**DOI:** 10.1039/d0na00772b

**Published:** 2020-10-19

**Authors:** Ali Ismael, Alaa Al-Jobory, Xintai Wang, Abdullah Alshehab, Ahmad Almutlg, Majed Alshammari, Iain Grace, Troy L. R. Benett, Luke A. Wilkinson, Benjamin J. Robinson, Nicholas J. Long, Colin Lambert

**Affiliations:** Department of Physics, Lancaster University Lancaster LA1 4YB UK k.ismael@lancaster.ac.uk; Department of Physics, College of Education for Pure Science, Tikrit University Tikrit Iraq; Department of Physics, College of Science, University of Anbar Anbar Iraq; Department of Chemistry, Imperial College London, MSRH White City London W12 0BZ UK

## Abstract

If the Seebeck coefficient of single molecules or self-assembled monolayers (SAMs) could be predicted from measurements of their conductance–voltage (*G*–*V*) characteristics alone, then the experimentally more difficult task of creating a set-up to measure their thermoelectric properties could be avoided. This article highlights a novel strategy for predicting an upper bound to the Seebeck coefficient of single molecules or SAMs, from measurements of their *G*–*V* characteristics. The theory begins by making a fit to measured *G*–*V* curves using three fitting parameters, denoted *a*, *b*, *c*. This ‘ABC’ theory then predicts a maximum value for the magnitude of the corresponding Seebeck coefficient. This is a useful material parameter, because if the predicted upper bound is large, then the material would warrant further investigation using a full Seebeck-measurement setup. On the other hand, if the upper bound is small, then the material would not be promising and this much more technically demanding set of measurements would be avoided. Histograms of predicted Seebeck coefficients are compared with histograms of measured Seebeck coefficients for six different SAMs, formed from anthracene-based molecules with different anchor groups and are shown to be in excellent agreement.

## Abstract 2

If the Seebeck coefficient of single molecules or self-assembled monolayers could be predicted from measurements of their conductance–voltage (*G*–*V*) characteristics alone, then the difficult experimental task of measuring their thermoelectric properties could be avoided.

## Graphical abstract

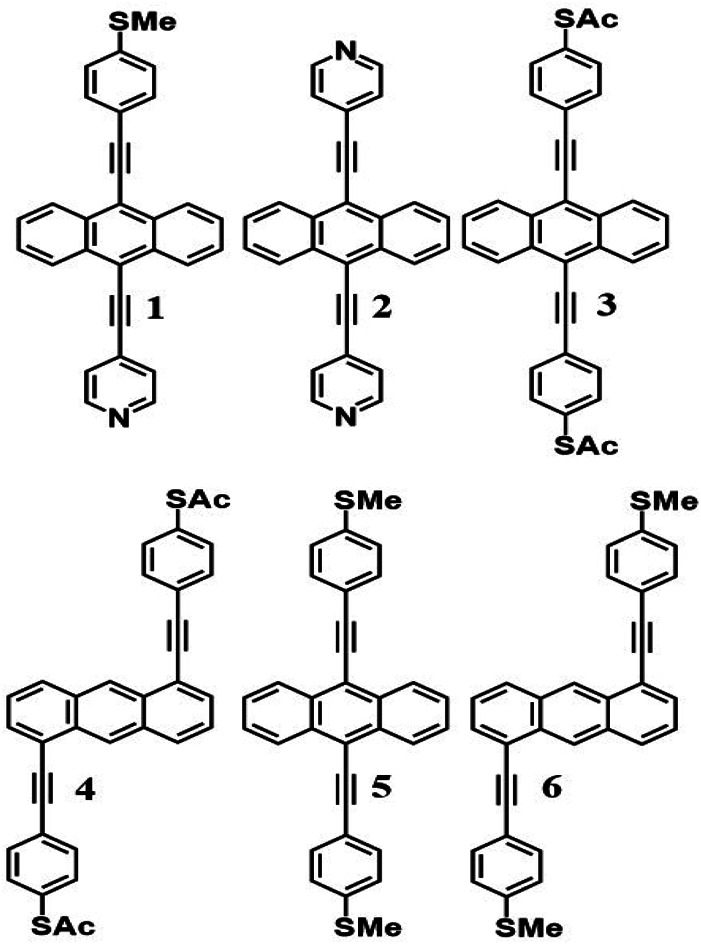

## Introduction

Recent studies of the thermoelectric properties of single molecules are motivated by the desire to probe fundamental properties of molecular-scale transport and by the desire to create high-performance thermoelectric materials using bottom-up designs.^1–3^ Following early experimental scanning thermopower microscope (STPM) measurements of the Seebeck coefficients of molecular monolayers,^4,5^ and early theoretical work^6^ suggesting that measurements of the Seebeck coefficient of a single molecule would provide fundamental information about the location of the Fermi energy of electrodes relative to frontier orbitals, Reddy *et al.*^7^ developed a modified scanning tunnelling microscope setup to measure the single-molecule Seebeck coefficient of a single molecule trapped between two gold electrodes. Although these and subsequent single-molecule measurements^8,9^ indeed yielded information about the Fermi energy, the resulting Seebeck coefficients were too low to be of technological significance. To address the problem of increasing the thermoelectric performance of organic molecules, Finch *et al.*^10^ demonstrated theoretically that large values of the Seebeck coefficient could be obtained by creating transport resonances and anti-resonances within the HOMO–LUMO gap and tuning their energetic location relative to the Fermi energy. Following these pioneering works, several experimental^11–20^ and theoretical studies^21–34^ have attempted to probe and improve the thermoelectric performance of single molecules. However, progress has been hampered by the additional complexity of thermoelectric measurement set-ups, because unlike measurements of single-molecule conductance, Seebeck measurements require additional control and determination of temperature gradients at a molecular scale. Due to this complexity, the number of experimental groups worldwide able to measure the Seebeck coefficient of single molecules is much lower than the number able to measure the conductance–voltage characteristics of single-molecules.

Herein we propose a simple and straightforward method of estimating an upper bound for the Seebeck coefficient of single molecules and self-assembled molecular layers (SAMs), based on measuring their conductance–voltage characteristics alone.

Since the latter are available to many experimental groups, this should speed up the screening of potential molecules for thermoelectric applications, without the need for direct measurement of their Seebeck coefficients. On the other hand, if the latter is also measured, then the proposed method enables a consistency check between measurements of complementary transport properties.

## ABC theory of molecular-scale thermoelectricity

Our starting point is the Landauer–Buttiker theory of phase-coherent transport, which utilises the transmission coefficient, *T*(*E*) describing the propagation of electrons of energy *E* from one electrode to the other *via* a single molecule or a SAM. A large body^35,36^ of experimental evidence suggests that when a molecule is placed between two metallic electrodes, the highest occupied molecular orbital (HOMO) and lowest unoccupied molecular orbital (LUMO) adjust themselves, such that the Fermi energy *E*_F_ of the electrodes lies within the HOMO–LUMO gap of the molecule. Furthermore, DFT simulations often reveal that the logarithm of the transmission function is a smooth function of energy near *E*_F_ and therefore it is reasonable to approximate *T*(*E*) by Taylor expansion of the form

1 ln *T*(*E*) = *a* + *b*(*E* − *E*_F_) + *c*(*E* − *E*_F_)^2^

In what follows, the coefficients *a*, *b*, *c* of this ‘ABC’ theory will be determined by fitting the above expression to measured low-voltage conductance–voltage curves, under the assumption that *a*, *b* and *c* do not change with voltage. Information about *T*(*E*) has been extracted from experimental measurements previously.^37,38^ ABC theory is aimed at describing off-resonance transport, since this is the most common case in molecular junctions and self-assembled monolayers. Of course, by applying an electrostatic of electrochemical gate, one could move transport towards resonance, but this is not relevant from the point of view of identifying thermoelectric materials. Our approach also applies to non-symmetric junctions, as demonstrated by molecule 1, and is not limited to the wide band approximation. In fact eqn (1) can describe many molecular junctions, but it could fail at high bias, because the proposed *I*–*V* fitting assumes that *I*–*V* curves are symmetric and therefore it should not be applied to junctions exhibiting strong rectification. However, it should be noted that the Seebeck effect is a low bias phenomenon, because typical values of the Seebeck coefficient are in the range of microvolts per Kelvin.

To acquire this fitting, we measure the current *versus* voltage at *M* different locations (labelled *j*) across a SAM. At each location, the current *I*^*j*^_exp_(*V*_*i*_) is measured at a series of *N* voltages labelled *V*_*i*_ between −1 V and +1 V, where *N* is typically several hundred. The corresponding conductance is defined to be *G*^*j*^_exp_(*V*_*i*_) = *I*^*j*^_exp_(*V*_*i*_)/*V*_*i*_. For each location *j*, we then computed the mean square deviations2
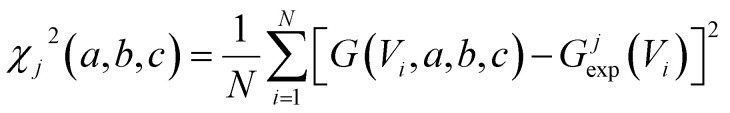


In this expression, *G*(*V*_*i*_, *a*, *b*, *c*) = *I*(*V*_*i*_, *a*, *b*, *c*)/*V*_*i*_ where *I*(*V*_*i*_, *a*, *b*, *c*) is the theoretical current obtained from the Landauer formula:3
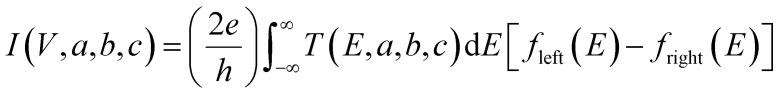
where *f*_left_(*E*) and *f*_right_(*E*) are the Fermi distributions of the left and right leads, with Fermi energies 
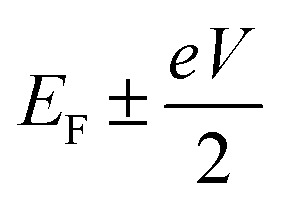
 respectively, *e* is the electronic charge, *h* is Planck's constant and *T*(*E*) is the transmission coefficient of eqn (1). The parameters *a*, *b*, *c* were then varied to locate the minimum of *χ*_*j*_^2^(*a*, *b*, *c*). The resulting values of *a*, *b*, *c* are denoted *a*_*j*_, *b*_*j*_, *c*_*j*_. From these fitted values, we obtained the predicted Seebeck coefficient for location *j* from the formula^30^4
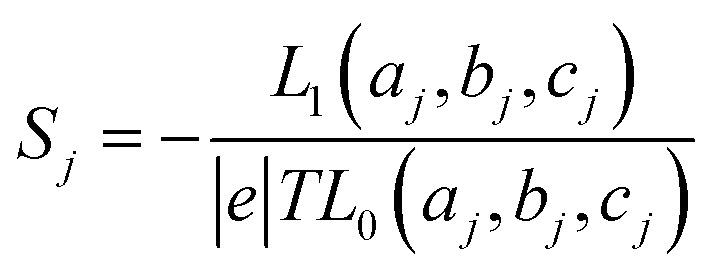
where5
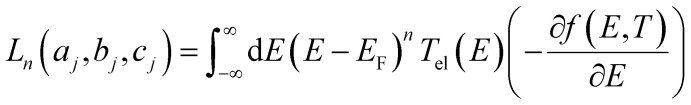
and *f*(*E*) = 1/[exp(*E* − *E*_F_)/*k*_B_*T* + 1] is the Fermi distribution. To demonstrate the validity of this ‘ABC’ theory, we then formed a histogram of these predicted values and compared these with histograms of experimentally measured Seebeck coefficients.

In fact, we found that in all cases, *c* was small and in many cases setting *c* = 0 yielded an acceptable fit. In what follows, we show results obtained by setting *c* = 0 and fitting *a* and *b* only. In Table S1 of the ESI,† we also show results obtained by allowing *c* to be non-zero.

It should be noted (see Section 3 of the ESI†) that ABC theory cannot predict the sign of the Seebeck coefficient, because it can only predict the magnitude of the coefficient *b*. To illustrate this point, note that at low-enough temperatures, the current *I* due to a source-drain bias voltage *V*, and the Seebeck coefficient *S* are given by6
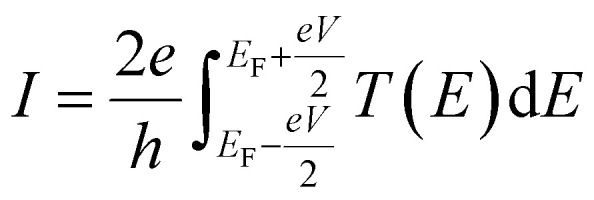
7
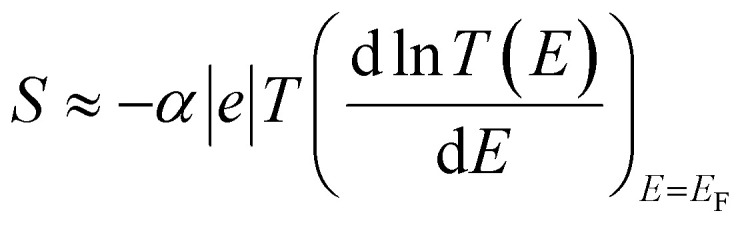
where *α* is the Lorentz number 
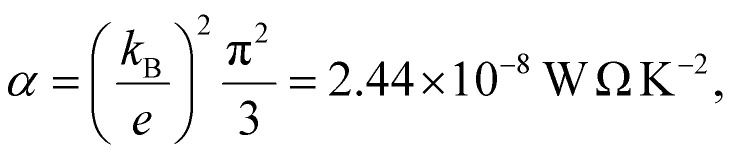
*e* is the electronic charge and *T* is the temperature.

This yields for the low-bias electrical conductance *G*,

8 *G* = *G*_0_*e*^*a*^

Assuming that an adequate fit can be obtained with *c* = 0, integration of eqn (6) yields9
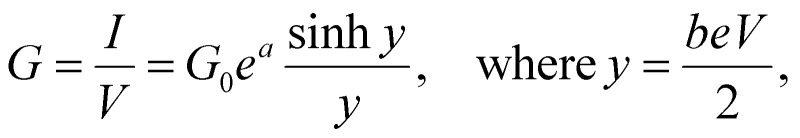
which reduces to eqn (8) in the limit *V* → 0. Alternatively, if *c* is non-zero, by differentiating eqn (6) one could fit to the differential conductance10
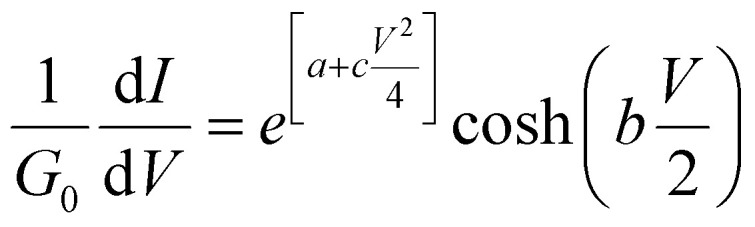


In Section 3 of the ESI,† it is demonstrated that the current *I*(*V*, *a*, *b*, *c*) in eqn (3) is an even function of *b*. This is also evident in the low-temperature eqn (9) and (10), since 
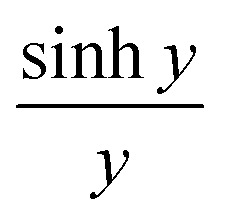
 and cosh *y* are even functions of *y*. Therefore, a fit to these formulae cannot determine the sign of *b*, because in eqn (2), *χ*_*j*_^2^(*a*, *b*, *c*) = *χ*_*j*_^2^(*a*, −*b*, *c*). In other words, if a minimum of *χ*_*j*_^2^(*a*, *b*, *c*) is found for a particular value of *b*, then there will also be a minimum at −*b*.

From eqn (7) and (1), this fitting yields the modulus of the Seebeck coefficient *via* the relation

11 |*S*| = −*α*|*e*||*b*|*T*

If *S* is a random variable, then the average of |*S*| is greater than or equal to the average of *S*. Therefore, from the average of |*S*|, ABC theory yields an upper bound for the average Seebeck coefficient.

Eqn (6) to (10) are valid at low temperatures only. At finite temperatures, the exact formula (3) is used to perform the fitting. In what follows, by simultaneously measuring both current–voltage relations and Seebeck coefficients, we demonstrate that ‘ABC’ theory indeed predicts an upper bound for the Seebeck coefficient from *I*–*V* curves.

## Results and discussion

Like all measurements of single molecule conductances and Seebeck coefficients in the literature, our results show a variety of *I*–*V* traces, which is why statistical analyses are required. These arise from variations in the geometries of molecules within the junction, variations in the shape of the electrode tip and variations in the manner in which a molecule attaches to an electrode.

**Fig. 1:**
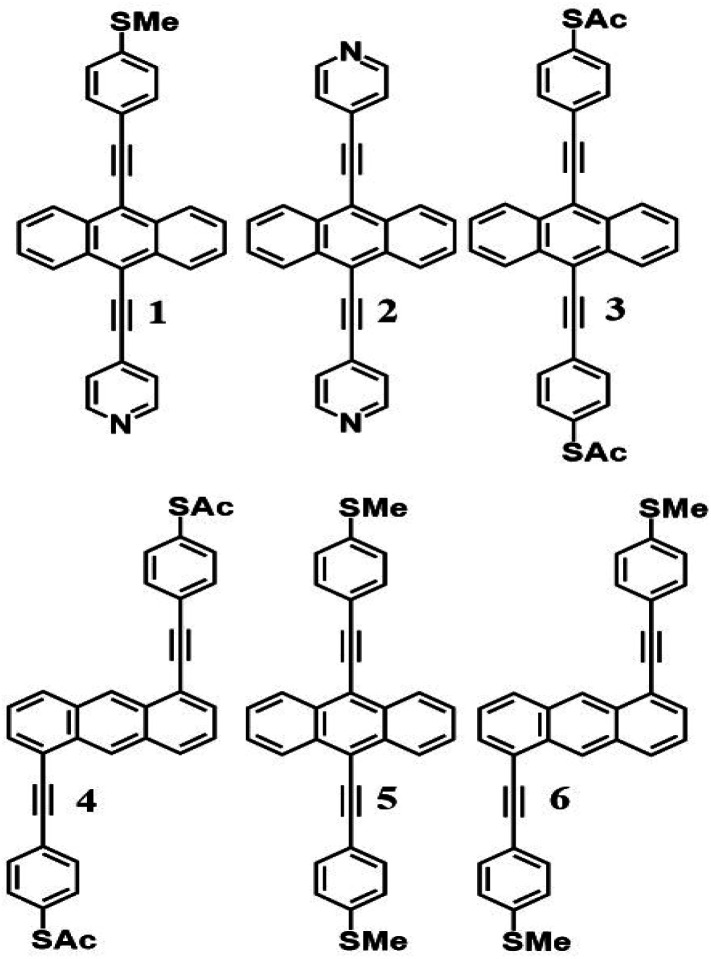
Structures of studied anthracene-based molecular wires. 1, 2, 3 and 5 correspond to the 7,2′ connectivity, while 4 and 6 correspond to the 1,5′ connectivity around the central anthracene core. These molecules also differ in the anchor groups through which they bind to a terminal electrode, with the binding groups denoted as follows; 1 = PySMe, 2 = 2Py, 3 and 4 = 2SAc, 5 and 6 = 2SMe.

**Fig. 2:**
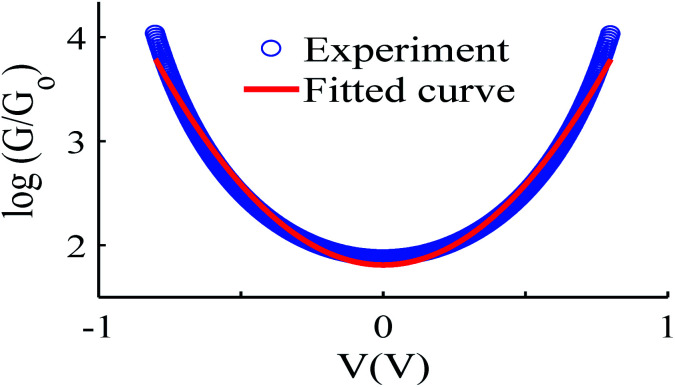
An example of the fitting process, experiment data (blue-circles) and fitted curve (red-solid line), also see curve fitting process in the ESI.†

We measured several hundred *I*–*V* curves for SAMs formed from the six anthracene-based molecules shown in Fig. 1, whose synthesis was reported previously,^39,40^ and then applied the above procedure to calculate the modulus of the Seebeck coefficient from each curve. Starting from a single raw *I*–*V* curve such as that shown in Fig. S8,† the ratio *G* = *I*/*V* was obtained (left panel of Fig. S9†) and the spike at zero volts was eliminated, as shown in the right-hand panel of Fig. S9.† Finally, we obtained a fitted curve from *I*–*V* data, as shown in Fig. S10.† The same procedure was repeated for each *I*–*V* curve of a given molecule. Fig. 2 shows a comparison between a fitted curve and the corresponding experimental data.

## Histograms of the Seebeck coefficients

**Fig. 3:**
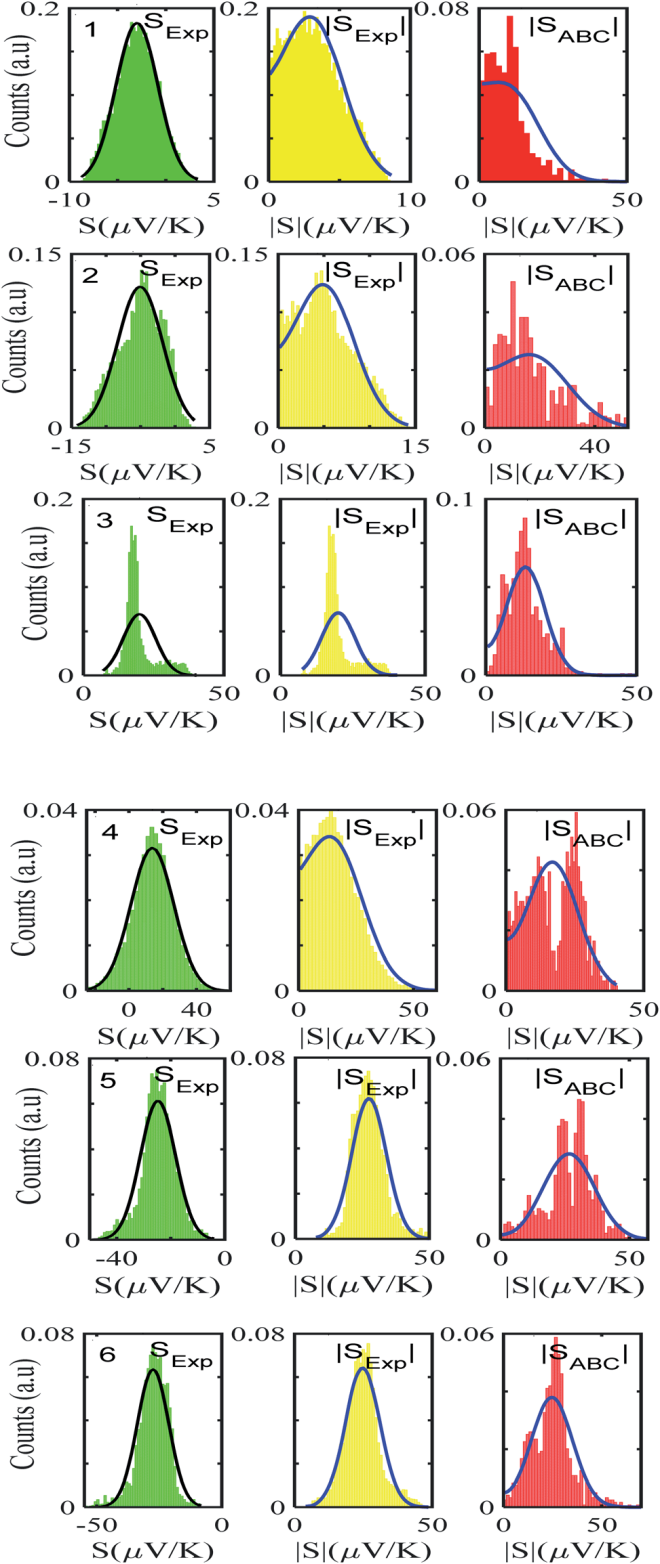
Experimentally derived and predicted ABC theory histograms along with their Gaussian and folded fit curves (black- and blue-solid lines) for molecules 1–6. Experiment, folded experiment and predicted ABC theory Seebeck coefficients (green, yellow and red, left to right).

After calculating the ‘*a*, *b*, *c*’ parameters from each single *I*–*V* curve, the corresponding |*S*| was obtained from eqn (4). These individual values were then used to construct a histogram of predicted |*S*| values for each molecule. These are the red histograms shown in Fig. 3 (see Fig. S11–S16,† for more details of the fitting process).

To demonstrate the validity of ABC theory, we also made many measurements of Seebeck coefficients at different locations across the SAM, and for each molecule, constructed a histogram of the resulting values. These are the green histograms of Fig. 3. These histograms were then ‘folded’ to yield the yellow histograms of experimental |*S*| values shown in Fig. 3 for each of the molecules 1–6.

**Fig. 4:**
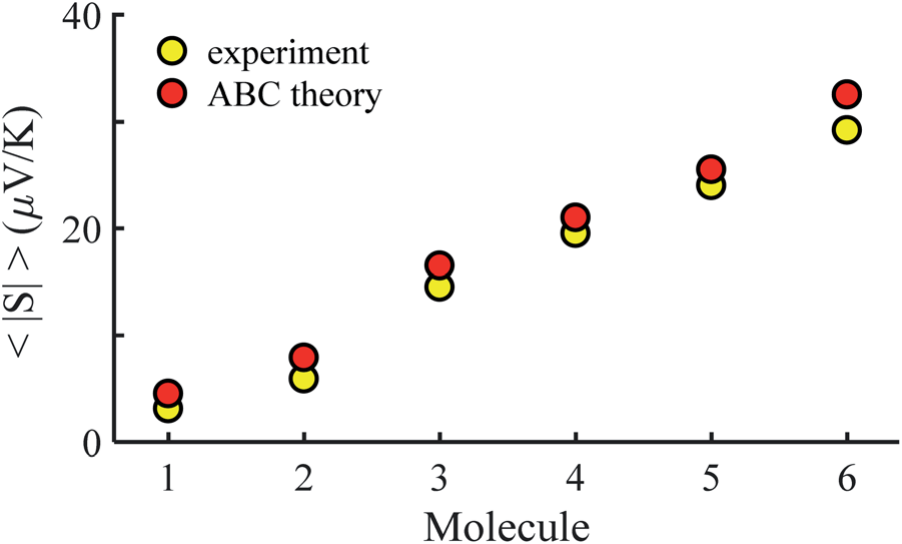
Experimental and ABC-theory predictions for average of the magnitudes of Seebeck coefficients 〈|*S*|〉 (yellow- and red-circles respectively).

Fig. 3 shows that the experimental and predicted histograms are in qualitative agreement. To make a quantitative comparison, we first computed the average |*S*| (denoted |*S*_ABC_|) from the red histograms and compared this with the average |*S*| (denoted |*S*_exp_| from the yellow histograms. These values are shown in Fig. 4, for each of the 6 molecules. This plot demonstrated strong overlap between experimental and ABC-theory values, clearly demonstrating the predictive ability of ABC theory. Our aim is to compare theory with experiment and since in the experimental histograms are fitted to a single Gaussian, we follow the same approach for the theoretical histograms. There are two peaks in the histograms of Fig. 3 (molecules 5 and 4). For molecule 5 these occur at |*S*| = 32.7 and |*S*| = 41.8 taking the average of these yields |*S*| = 37.2 which is very close to our quoted value for the most-probable |*S*| (*i.e.* |*S*| = 37.3). Similarly, for molecule 4 these occur at |*S*| = 11.4 and |*S*| = 24.3 taking the average of these yields |*S*| = 17.8, which is close to our quoted value (|*S*| = 17.5). Therefore fitting to a single Gaussian provides an adequate prediction for |*S*| for the studied molecules.

The averages in Fig. 4 were obtained by making a Gaussian fit to the experimentally-measured (green) histograms, as is common practice in the literature. If each of the green histograms of measured values of *S* is assumed to approximate a Gaussian distribution of the form12
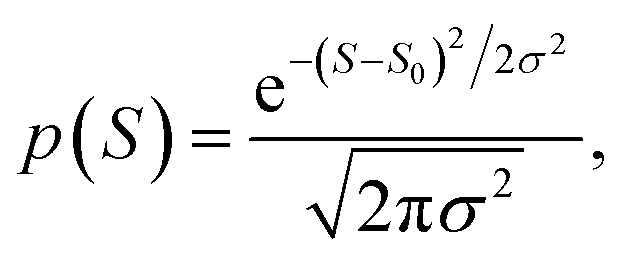
where *S*_0_ is the average of *S*, and *σ* is the standard deviation, then 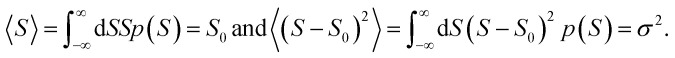 This means that measured values of |*S*| possess a folded Gaussian distribution of the form *f*(|*S*|) = *p*(|*S*|) + *p*(−|*S*|). *i.e.*13
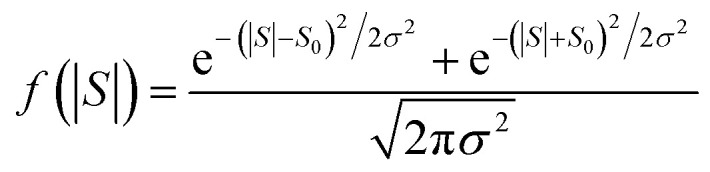
or equivalently14
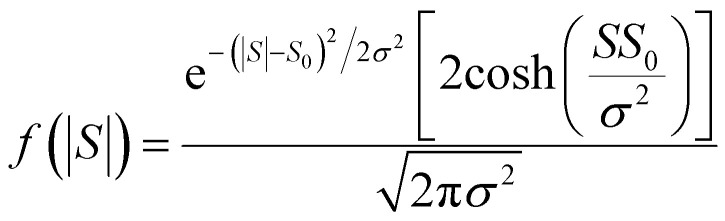


For |*S*_0_| < *σ*, *f*(|*S*|) has a maximum at |*S*| = 0, whereas for |*S*_0_| > *σ*, the maximum occurs at |*S*| ≠ 0. The blue curves in Fig. 3 show a fit of this function to each of the red histograms. The black curves show plots of the corresponding Gaussian distributions. For the experimental averages corresponding to the yellow points in Fig. 4 and for the ABC-predicted averages corresponding to the red points in Fig. 4, the average was computed by fitting a folded Gaussian *f*(|*S*|) to the histogram of predicted values of |*S*| and then using the formula
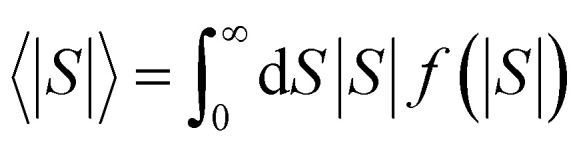


**Fig. 5:**
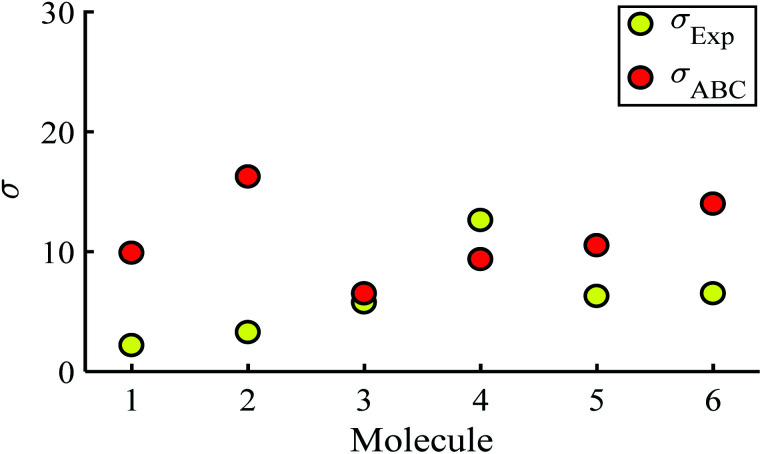
Standard deviations *σ* obtained from experiment and predicted ABC theory data (yellow- and red-circles).

Fig. 5 shows a comparison between the resulting *σ* values for each of the molecules, obtained by fitting eqn (13) to the red histograms and by fitting eqn (12) to the green histograms. This shows that qualitative information about the widths of the distributions can also be obtained from ABC theory. Fig. 5 shows similar results for the ABC standard deviations *σ*_ABC_ and the experimental *σ*_Exp._ for most molecules, whereas there is a larger difference for molecules 1 and 2. To address this point, the distributions of the root mean square deviations *χ*_*i*_ (see eqn (2)) from each individual *G*–*V* fit (labelled *i*), for the 6 molecules, are shown in Fig. S24.† The mean values 〈*χ*〉 of these values of *χ*_*i*_ are shown in Table S3† for each molecule. This shows that molecule 2 has the largest root mean square deviations 〈*χ*〉 = 1.5 × 10^−2^ and this corresponds to the largest difference Δ*σ* = *σ*_ABC_ − *σ*_Exp._ between standard deviations of the theory and experiment. Similarly, molecule 1 has the next highest value of 〈*χ*〉 and the next highest value of Δ*σ*. Molecule 3 has the lowest value of Δ*σ* and the lowest value of 〈*χ*〉. This correlation between 〈*χ*〉 and Δ*σ* is shown more clearly in Fig. S25† and demonstrates that the fitting parameter 〈*χ*〉 is an indicator of the accuracy of the predicted value of |*S*| made by ABC theory.

It is worth mentioning that in the above analysis, Seebeck coefficients have been calculated by fitting to *G*–*V* curves rather than *I*–*V* curves. Table S2† shows a comparison between the results obtained from *I*–*V* fits and *G*–*V* fits for twelve different sets of *I*–*V* measurements and show that the results are comparable.

## Conclusion

By making simultaneous measurements of the Seebeck coefficients and conductance–voltage characteristics of SAMs formed from six anthracene-based molecules with different anchor groups, we have demonstrated that ‘ABC’ theory allows for the prediction of magnitudes of Seebeck coefficients by making fits to measured conductance–voltage relations using three fitting parameters, denoted *a*, *b*, *c*. This is advantageous because it means that by measuring the *G*–*V* characteristics of single molecules or SAMs, their potential for high-performance thermoelectricity can be assessed without the need for experimentally derived Seebeck coefficients. In addition to this, if measurements of the latter are available, then ‘ABC’ theory can be applied as a consistency check between the two sets of measurements. The theory presented within this work represents an important step forward in the study of molecular thermoelectrics, greatly easing accessibility of the field to those without access to the specialist equipment usually needed to perform such complex thermal measurements.

## Author contributions

C. J. L. and A. K. I. conceived the concept. A. A., A. A., M. A. and A. A. carried out the calculations. X. W. and B. R. performed the *I*–*V* measurements. N. J. L., T. L. R. B. and L. A. W. synthesised the molecules. All co-authors assisted in writing the manuscript. C. J. L. and A. K. I. supervised the research and provided essential contributions to interpreting the results and drafting the manuscript.

## Conflicts of interest

There are no conflicts to declare.

## Supplementary Material

NA-002-D0NA00772B-s001
