# Correction: Molecular-scale thermoelectricity: as simple as ‘ABC’

**DOI:** 10.1039/d0na90067b

**Published:** 2021-01-11

**Authors:** Ali Ismael, Alaa Al-Jobory, Xintai Wang, Abdullah Alshehab, Ahmad Almutlg, Majed Alshammari, Iain Grace, Troy L. R. Bennett, Luke A. Wilkinson, Benjamin J. Robinson, Nicholas J. Long, Colin Lambert

**Affiliations:** Department of Physics, Lancaster University Lancaster LA1 4YB UK k.ismael@lancaster.ac.uk; Department of Physics, College of Education for Pure Science, Tikrit University Tikrit Iraq; Department of Physics, College of Science, University of Anbar Anbar Iraq; Department of Chemistry, Imperial College London, MSRH White City London W12 0BZ UK

## Abstract

Correction for ‘Molecular-scale thermoelectricity: as simple as ‘ABC’’ by Ali Ismael *et al., Nanoscale Adv.*, 2020, **2**, 5329–5334, DOI: 10.1039/D0NA00772B.

The authors regret that the name of one of the authors (Troy L. R. Bennett) was shown incorrectly in the original article. The corrected author list is as shown above.

The Royal Society of Chemistry apologises for these errors and any consequent inconvenience to authors and readers.

## Supplementary Material

