# Supplementary material for: Molecular-scale thermoelectricity: as simple as ‘ABC’
Source: Nanoscale Adv. 2020 Oct 19;2(11):5329–34. doi: 10.1039/d0na00772b (PMC9417915; doi:10.1039/d0na00772b)
Supplement: NA-002-D0NA00772B-s001 [file NA-002-D0NA00772B-s001.pdf]

Electronic Supplementary Material (ESI) for Nanoscale Advances.
This journal is © The Royal Society of Chemistry 2020

Supplementary Information

Molecular-scale thermoelectricity: as simple as 'ABC'.

Ali Ismael[a,b,*], Alaa Al-Jobory [a,c], Xintai Wang[a], Abdullah Alshehab[a], Ahmad Almutlg[a], Majed Alshammari[a], Iain Grace[a], Troy L. R. Bennett[d], Luke A. Wilkinson[d], Benjamin Robinsona[a], Nicholas J. Long[d] and Colin Lambert[a,*]

[a] Physics Department, Lancaster University, Lancaster, LA1 4YB, UK.

[b] Department of Physics, College of Education for Pure Science, Tikrit University, Tikrit, Iraq.

[c] Department of Physics, College of Science, University Of Anbar, Anbar, Iraq.

[d] Department of Chemistry, Imperial College London, MSRH, White City, London, W12 0BZ.

*To whom correspondence should be addressed. e-mail: k.ismael@lancaster.ac.uk; and c.lambert@lancaster.ac.uk;

Table of Contents:

Theory calculations

1. Molecules used in this study S2
2. Experiment data ($I-V$ characterisation) S2
3. Proof that $I(V,a,b,c) = I(V,a,-b,c)$ S6
4. Curve fitting S6
5. Curve fitting for molecules of study S8
6. Histogram of Seebeck coefficient S11
7. Histogram fitting S13
8. Seebeck coefficient c non-zero (c$\neq$ 0) S14
9. Seebeck coefficient obtained from $\boldsymbol{I-V}$ fit versus $\boldsymbol{G}$ fit S14
10. Mean square deviations $\boldsymbol{\chi}$ versus standard deviations σ S15

Experiment measurements

11. Histogram S17

References

1. Molecules used in this study

In this study the 6 molecules were selected, the structures of which are shown in Fig. S1. These systems demonstrate both positive and negative Seebeck coefficients and were selected to compare their measured Seebeck values compared to those predicted by the ABC model.

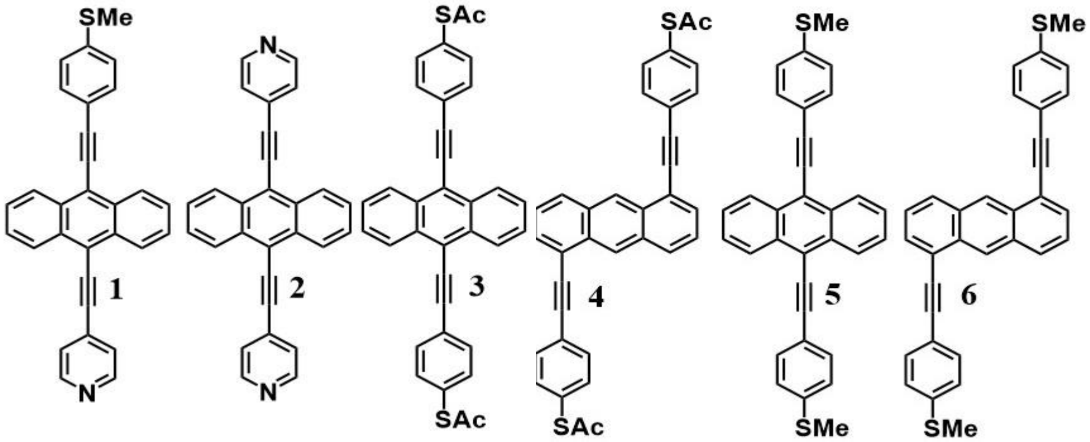

Figure S1: Structures of the molecules studied in this work (synthesis reported previously)[35, 36]

2. Experiment data ($I - V$ characterisation)

Molecular conductance was characterized by conductive AFM (cAFM). Single molecule equivalence values were calculated by dividing the total conductance by the number of molecules in the junction, where the number of molecules contacted by the probe was calculated using contact area between sample and probe dividing the occupation area of a single molecule derived from QCM. The contact area between sample and probe was estimated by Hertzian model:

$$\mathrm{r} = (\mathrm{F} \times \mathrm{R} \times \frac{1}{\mathrm{Y}})^{\frac{1}{3}}$$

$$\frac{1}{\mathrm{Y}} = \frac{3}{4} \times (\frac{1 - \mathrm{v}_1^2}{\mathrm{E}_1} + \frac{1 - \mathrm{v}_2^2}{\mathrm{E}_2})$$

where r the contact radius, F the loading force from probe to sample, R the radius of the probe (~18 nm from the supplier), v_1 and v_2 the Poisson ratio of the material, E_1 and E_2 the Young's Modulus for probe (~ 100 GPa) and SAMs (~10 GPa).

The electrical transport properties of the SAMs were characterized using a custom cAFM system. The cAFM setup is based on a multi-mode8 AFM system (Bruker nanoscience). The bottom gold substrate was used as the source, and a Pt/Cr coated probe (Spark 70Pt, Nunano Ltd) was used as the drain. The force between probe and molecule was controlled at 2 nN, as this force is strong enough for the probe to penetrate through the water layer on the sample surface but not too strong so as to destroy the molecular thin film. The driven bias was added between the source and drain by a voltage generator (Aglient 33500B), the source to drain current was amplified by a current pre-amplifier (SR570, Stanford Research Systems), and the IV characteristics of the sample was collected by the computer. In this section, nearly a thousand $I - V$ curves were collected for each molecule, examples of which are shown in Figs. S2-S7.

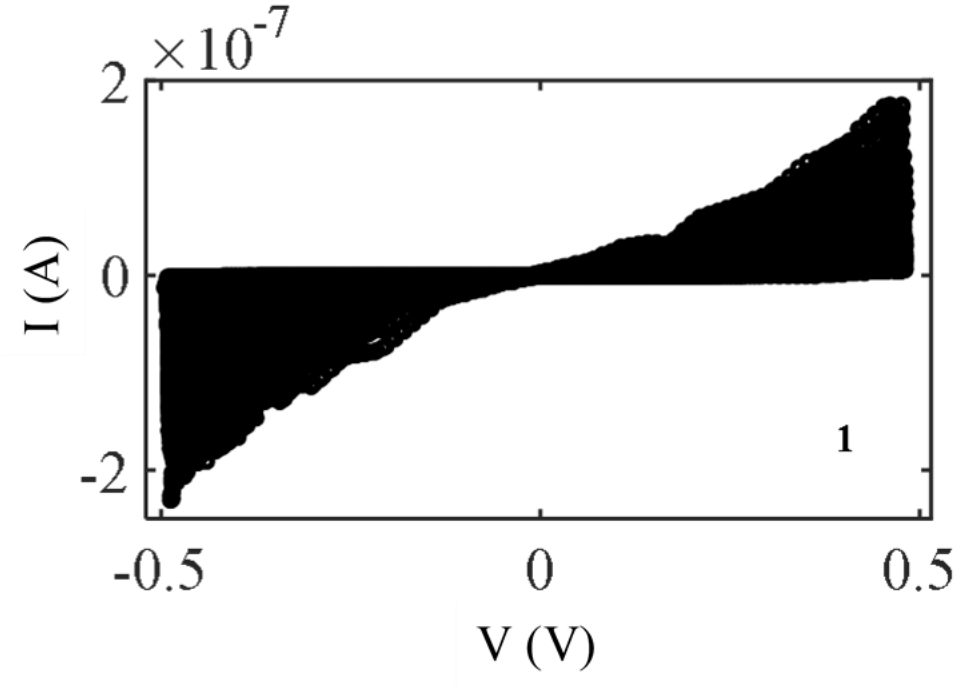

Figure S2: $I - V$ curves for **1.**

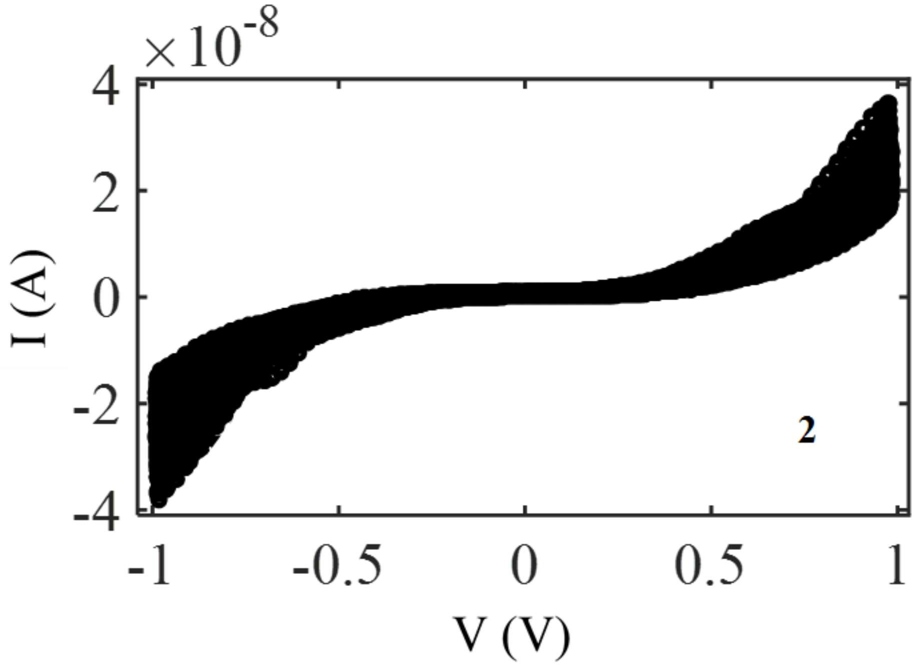

Figure S3: $I - V$ curves for **2**.

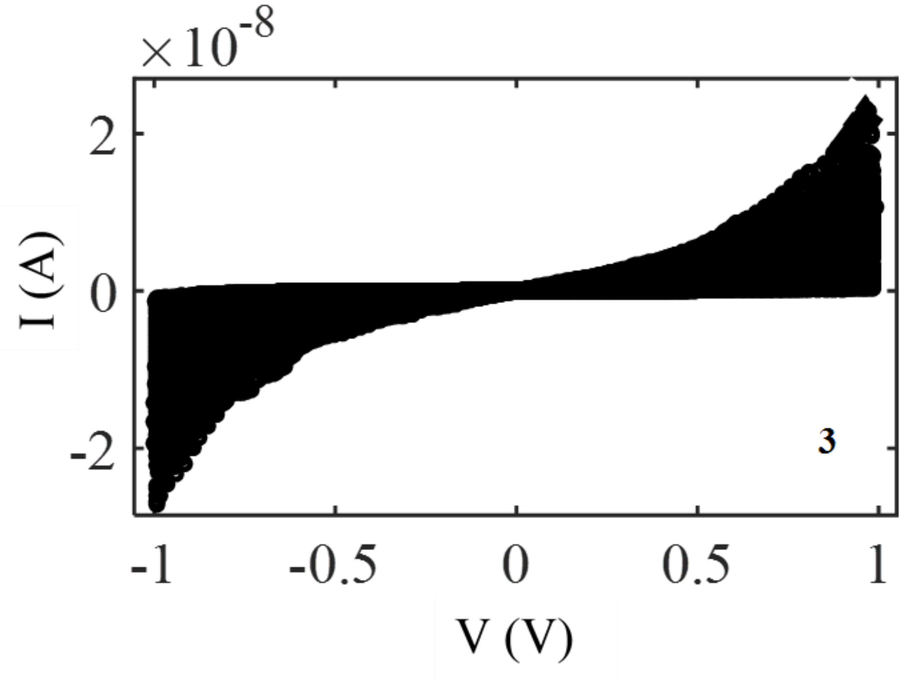

Figure S4: $I - V$ curves for **3.**

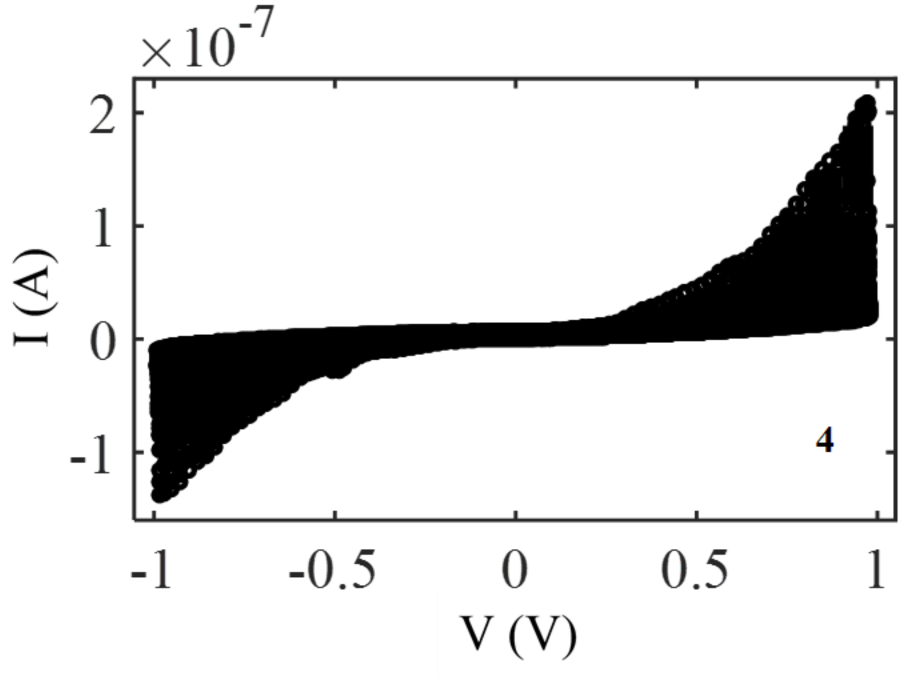

Figure S5: $I - V$ curves for **4**.

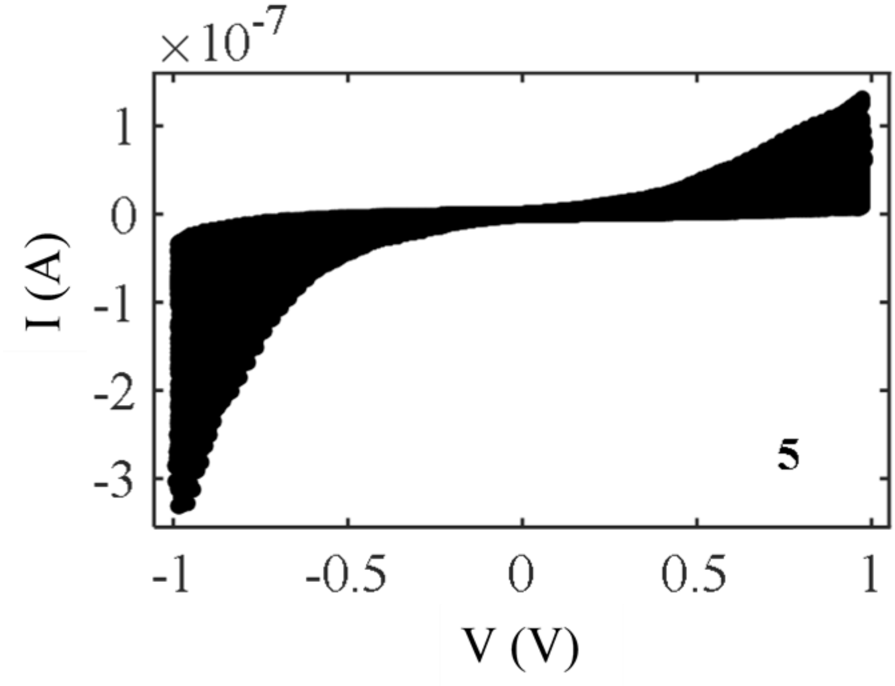

Figure S6: $I - V$ curves for **5**.

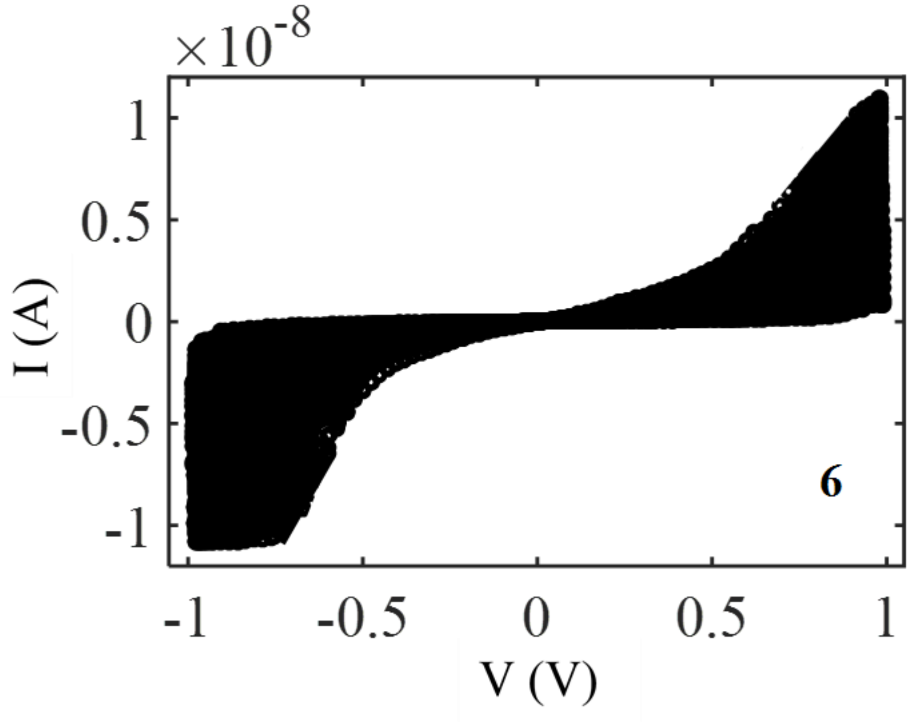

Figure S7: $I - V$ curves for **6**.

3. Proof that $\boldsymbol{I(V,a,b,c) = I(V,a,-b,c)}$

From equations (1) and (3) of the main text

$$I(V,a,b,c) = \left(\frac{2e}{h}\right)\int_{-\infty}^{\infty} dx e^{(a+bx+cx^2)} \left[\frac{1}{e^{\frac{x-v}{k_BT}}+1} - \frac{1}{e^{\frac{x+v}{k_BT}}+1}\right] \quad \text{(S.1)}$$

where $x = E - E_F$ and $v = eV/2$. ie

$$I(V,a,b,c) = \left(\frac{2e}{h}\right)\int_{-\infty}^{\infty} dx e^{(a+bx+cx^2)} \left[\frac{e^{\frac{x+v}{k_BT}}-e^{\frac{x-v}{k_BT}}}{\left[e^{\frac{x-v}{k_BT}}+1\right][e^{\frac{x+v}{k_BT}}+1]}\right] \quad \text{(S.2)}$$

After making the substitution $y = -x$ and replacing b by $-b$, equation (S.1) becomes

$$I(V,a,-b,c) = \left(\frac{2e}{h}\right)\int_{+\infty}^{-\infty} (-dy) e^{(a+by+cy^2)} \left[\frac{1}{e^{\frac{-y-v}{k_BT}}+1} - \frac{1}{e^{\frac{-y+v}{k_BT}}+1}\right]$$

Ie

$$I(V,a,-b,c) = \left(\frac{2e}{h}\right)\int_{-\infty}^{+\infty} dy e^{(a+by+cy^2)} \left[\frac{e^{\frac{y+v}{k_BT}}}{e^{\frac{y+v}{k_BT}}+1} - \frac{e^{\frac{y-v}{k_BT}}}{e^{\frac{y-v}{k_BT}}+1}\right]$$

ie

$$I(V,a,-b,c) = \int_{-\infty}^{+\infty} dy e^{(a+by+cy^2)} \left[\frac{e^{\frac{y+v}{k_BT}}-e^{\frac{y-v}{k_BT}}}{[e^{\frac{y+v}{k_BT}}+1][e^{\frac{y-v}{k_BT}}+1]}\right] \quad \text{(S.3)}$$

Since equations (S.2) and (S.3) are identical, this completes the proof and demonstrates that only $|b|$ can be predicted by ABC theory.

4. Curve fitting

We gathered several hundred $\boldsymbol{I-V}$ curves for each molecule utilising an STM device, and then applied the fitting procedure described in the main text to calculate the modulus of the corresponding Seebeck coefficients. Figure S8 shows an example of a single raw $\boldsymbol{I-V}$ curve. The left panel of Fig. S9 shows the ratio $\boldsymbol{I/V}$ used to obtain the finite-voltage conductance $\boldsymbol{G}$. After eliminating the spike close to zero voltage (red-dashed rectangular), the resulting $\boldsymbol{G-V}$ curve is shown in right panel of Fig. S9. Fig. S10 shows the fitted curve from $\boldsymbol{G-V}$ data.). This process is applied to individual $\boldsymbol{I-V}$ curves of each molecule within this study (**1-6**).

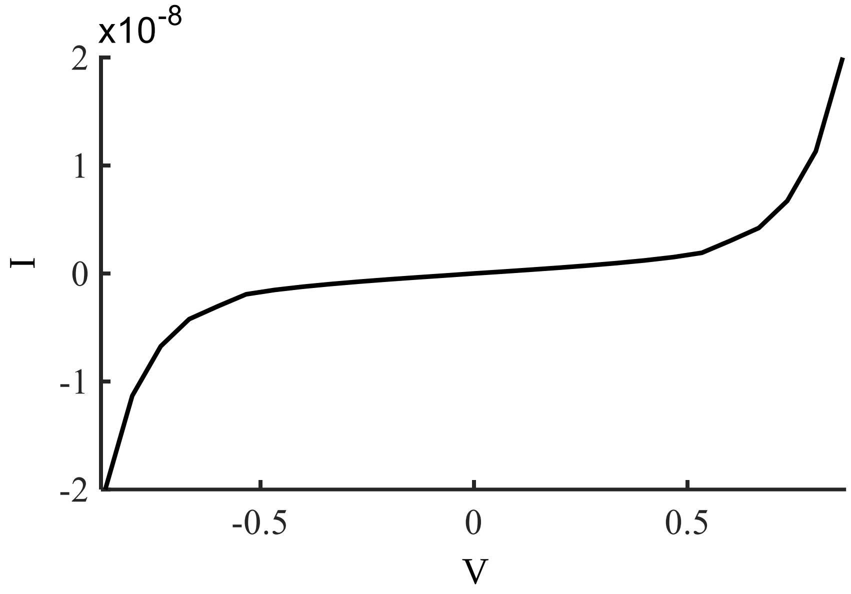

Figure S8: An example of an $I - V$ curve

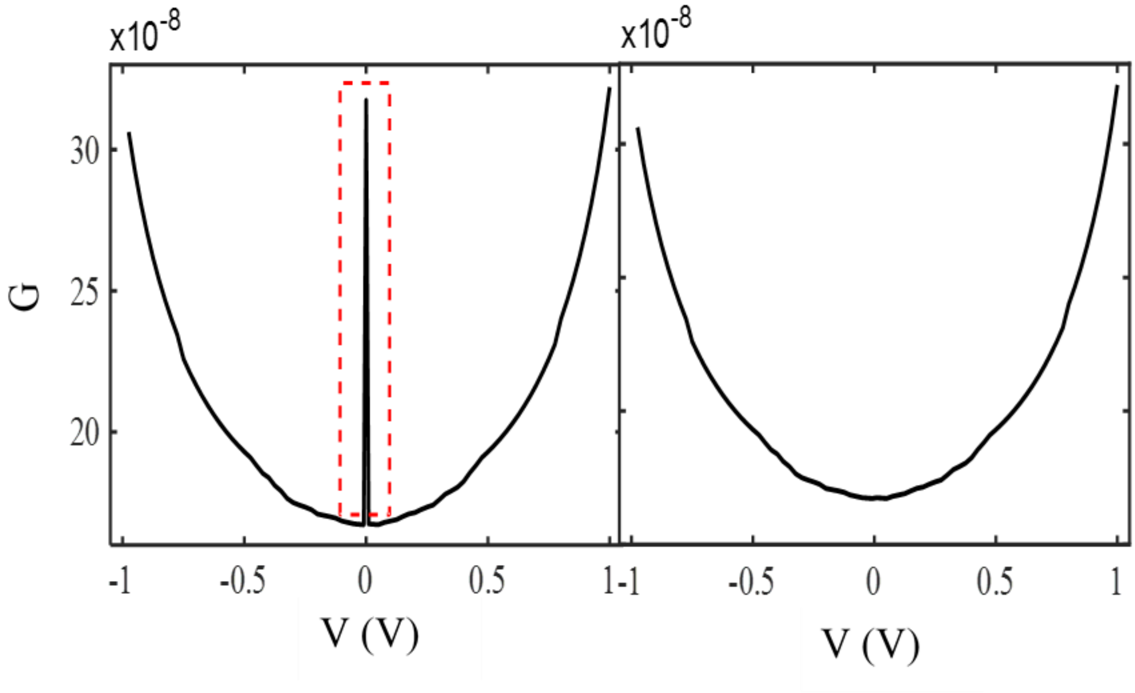

Figure S9: (Left panel) Experimental $G - V$ data. (Right panel) The same curve after deleting the spike close to zero volts.

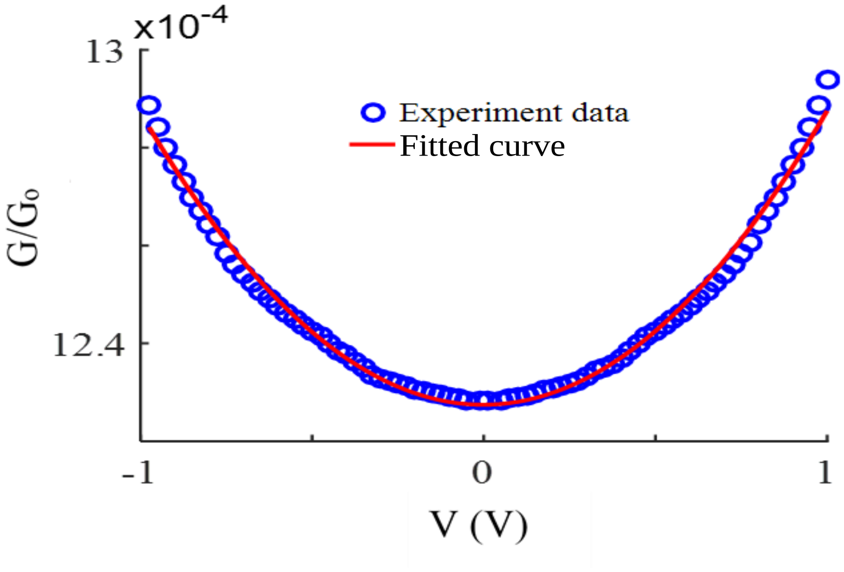

Figure S10: A comparison between experimental values of G/G_0 versus voltage V(blue-circles) and the fitted curve from ABC theory (red-solid line).

5. Curve fitting for molecules studied

Here, we present an example of fitting to an $\boldsymbol{I - V}$ curve for each molecule. This was achieved using the MATLAB routine 'FIT' to find the minimum of $\boldsymbol{\chi_j^2(a, b, c)}$, defined in equation (2).

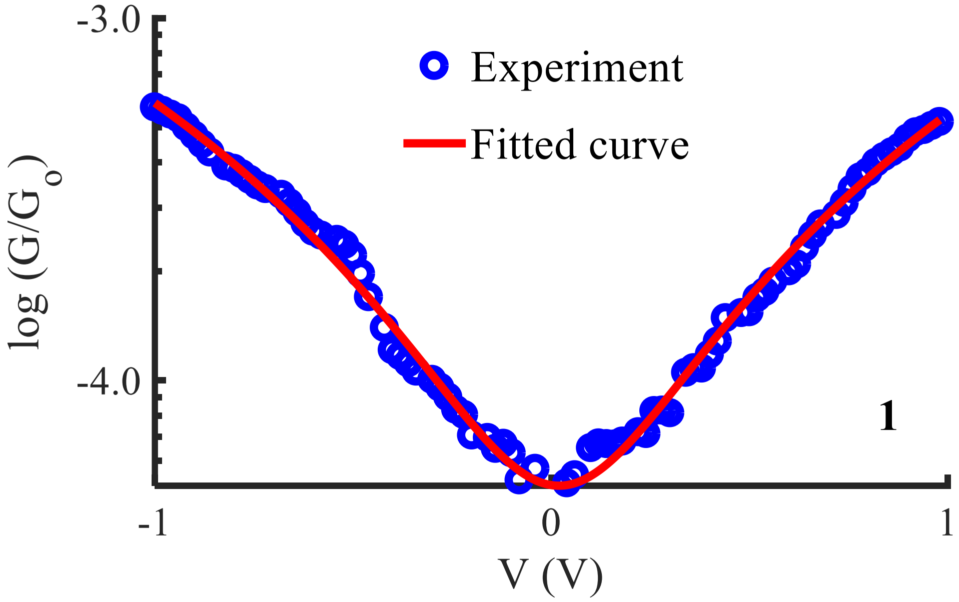

Figure S11: A comparison between experimental values of $log\ (G/G_0)$ versus voltage V(blue-circles) and the fitted curve from ABC theory (red-solid line) for molecule **1**.

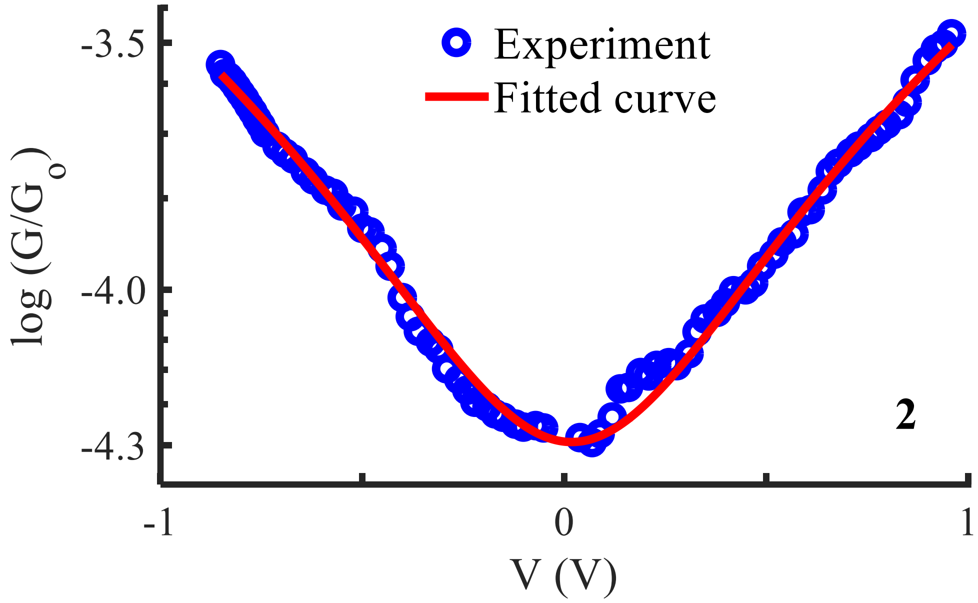

Figure S12: A comparison between experimental values of $log\ (G/G_0)$ versus voltage V(blue-circles) and the fitted curve from ABC theory (red-solid line) for molecule **2**.

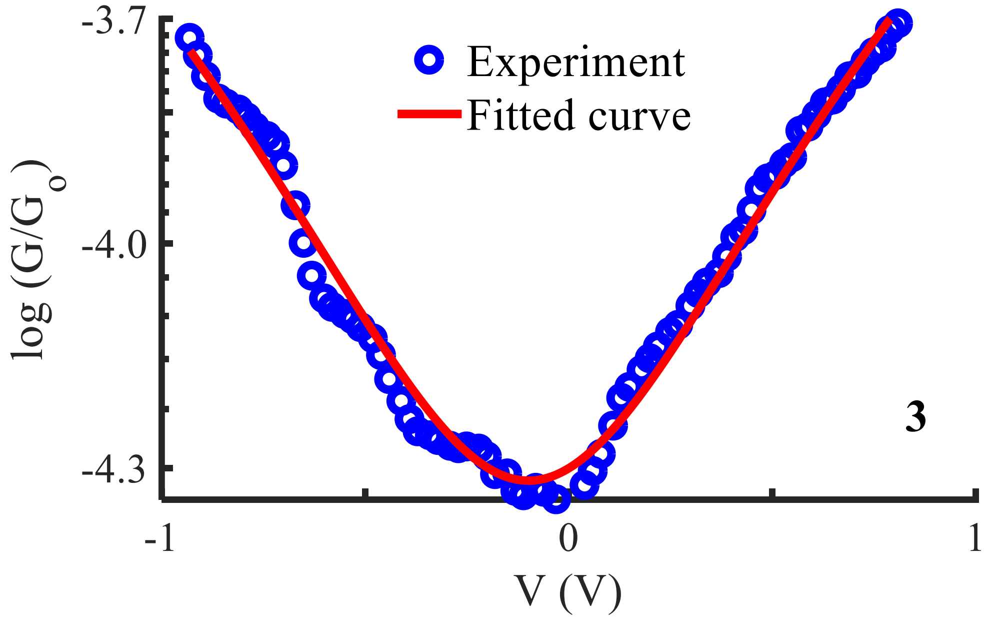

Figure S13: A comparison between experimental values of $log\ (G/G_0)$ versus voltage V(blue-circles) and the fitted curve from ABC theory (red-solid line) for molecule **3**.

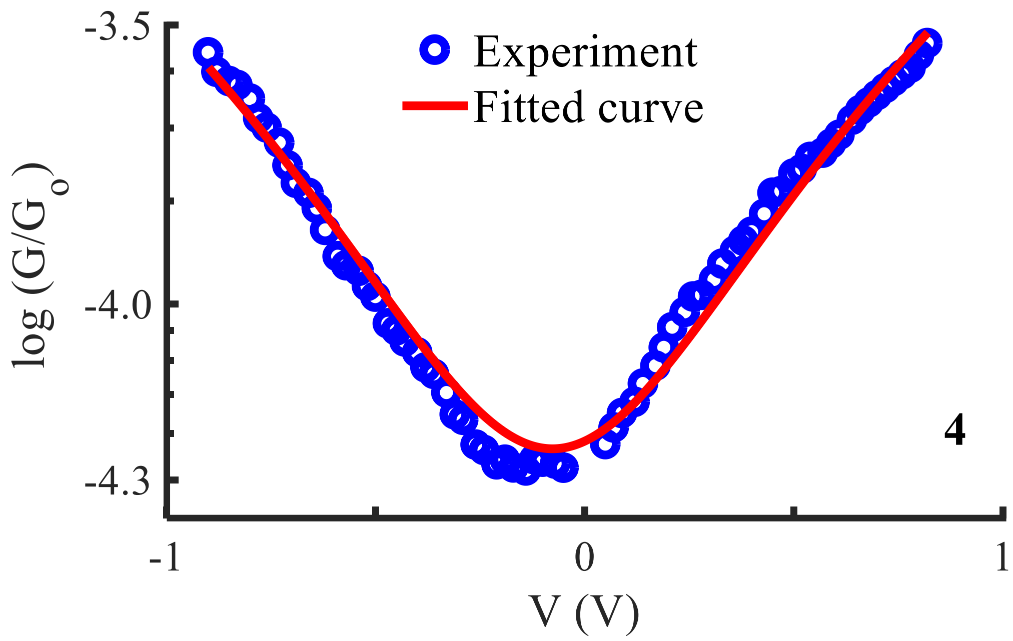

Figure S14: A comparison between experimental values of $log\ (G/G_0)$ versus voltage V(blue-circles) and the fitted curve from ABC theory (red-solid line) for molecule **4**.

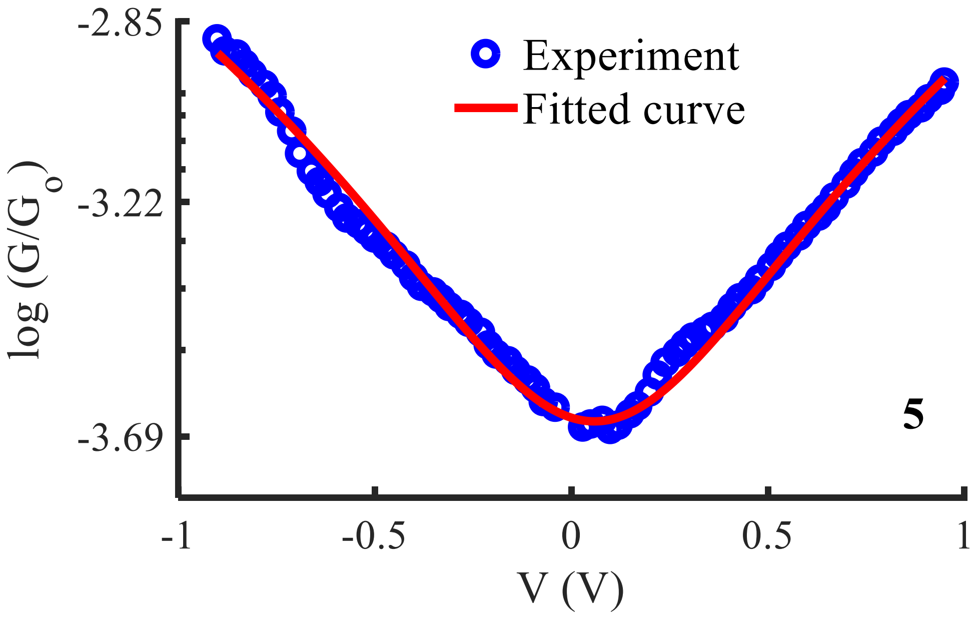

Figure S15: A comparison between experimental values of $log\ (G/G_0)$ versus voltage V(blue-circles) and the fitted curve from ABC theory (red-solid line) for molecule **5**.

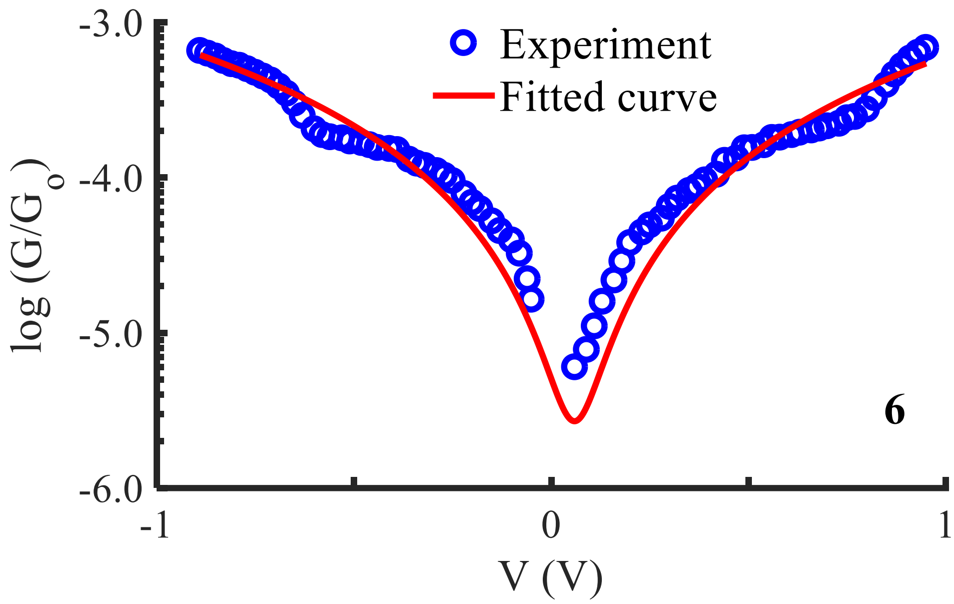

Figure S16: A comparison between experimental values of $log\ (G/G_0)$ versus voltage V(blue-circles) and the fitted curve from ABC theory (red-solid line) for molecule **6**.

6. Histograms of Seebeck coefficients

Experimental measurements provide histograms for molecules **1-6** (green histograms of Figs S17-S22). By applying the fitting process on each $G - V$ curve and using Equ.4 we obtain the Seebeck coefficient values. From that data, a histogram has been generated for each molecule (red histograms of Figs S17-S22), from the $I - V$ experiment data (see Figs S2-S8). It is worth mentioning that the 'ABC' model predicts the absolute value of the Seebeck histogram $|S|$, whereas experimentally-measured Seebeck histograms, which could be positive or negative as shown in the green histograms of Figs S17-S22. To compare theory predicted Seebeck histograms against experimentally-measured Seebeck histogram the absolute value has been taken for the experiment results (yellow histograms). Figures S17-S22 show a comparison between the experiment and theory absolute histograms $|S|$ (yellow and red histograms).

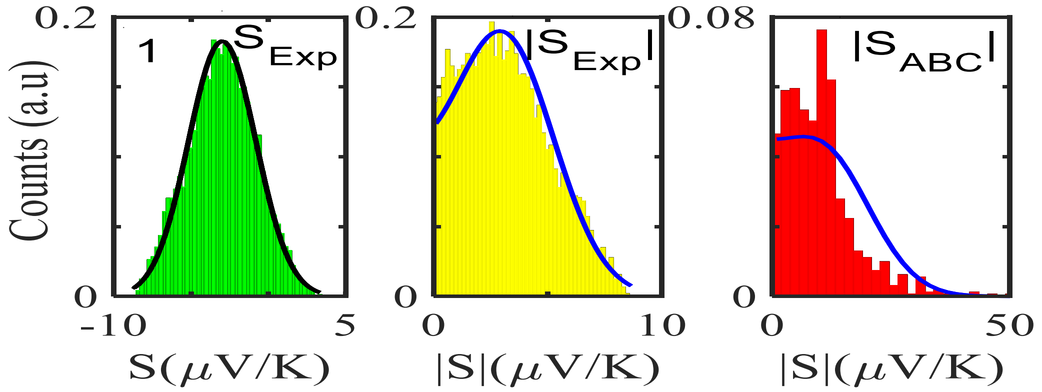

Figure S17: Experiment and theory histograms along with their Gaussian and folded fit curves (black- and blue-solid lines) for **1** (green experiment, yellow absolute experiment and red absolute theory)

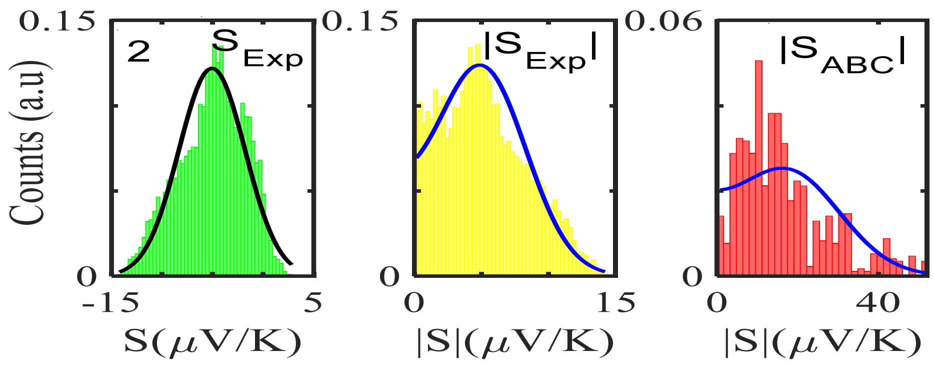

Figure S18: Experiment and theory histograms along with their Gaussian and folded fit curves (black- and blue-solid lines) for **2** (green experiment, yellow absolute experiment and red absolute theory)

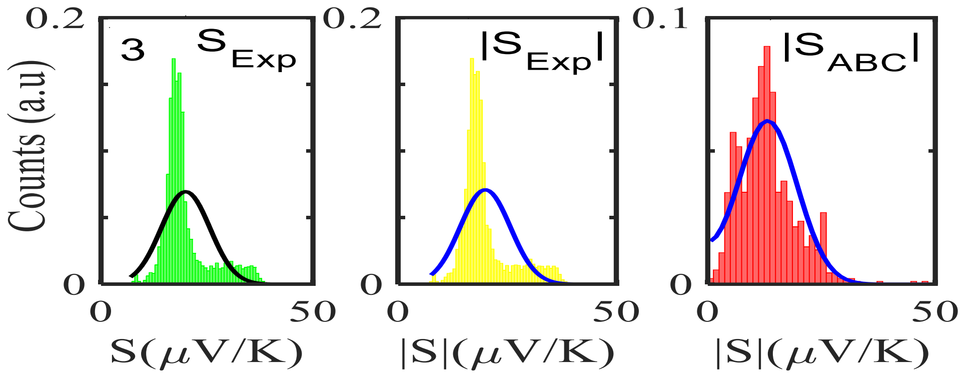

Figure S19: Experiment and theory histograms along with their Gaussian and folded fit curves (black- and blue-solid lines) for **3** (green experiment, yellow absolute experiment and red absolute theory)

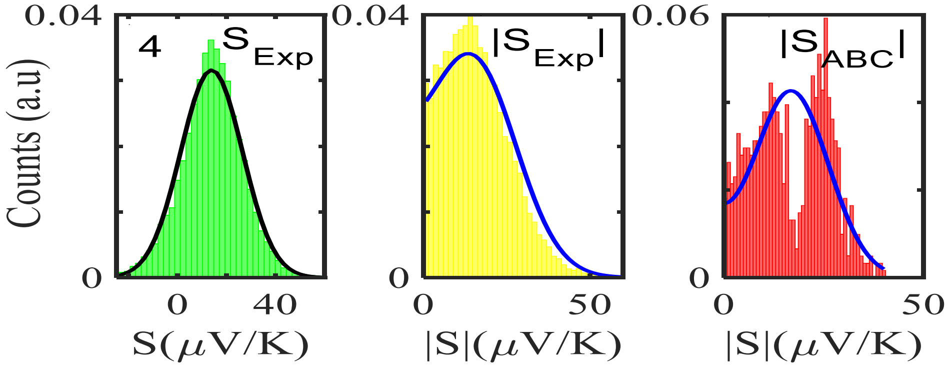

Figure S20: Experiment and theory histograms along with their Gaussian and folded fit curves (black- and blue-solid lines) for **4** (green experiment, yellow absolute experiment and red absolute theory)

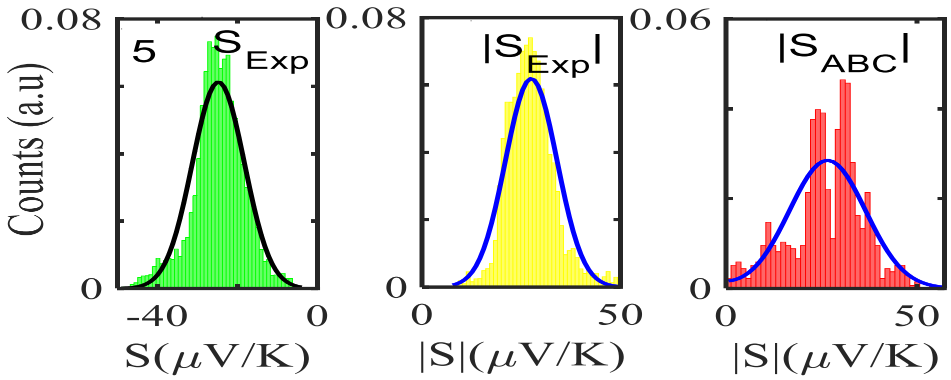

Figure S21: Experiment and theory histograms along with their Gaussian and folded fit curves (black- and blue-solid lines) for **5** (green experiment, yellow absolute experiment and red absolute theory)

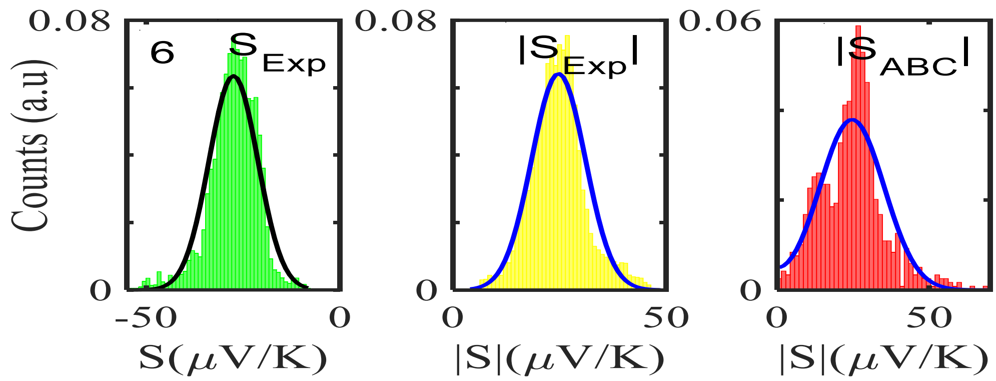

Figure S22: Experiment and theory histograms along with their Gaussian and folded fit curves (black- and blue-solid lines) for **6** (green experiment, yellow absolute experiment and red absolute theory)

7. Histogram fitting

In section 7, the experimental and theoretical histograms S and $|S|$ are plotted. Gaussian fit has been made for all histograms (black-sold line). Figure S23, shows the experimentally-measured Seebeck S (green-circles), the absolute experimentally-measured Seebeck $|S|$ (yellow -circles) and the absolute predicted Seebeck $|S|$(red-circles) along with their folded fit curves (blue-sold line)

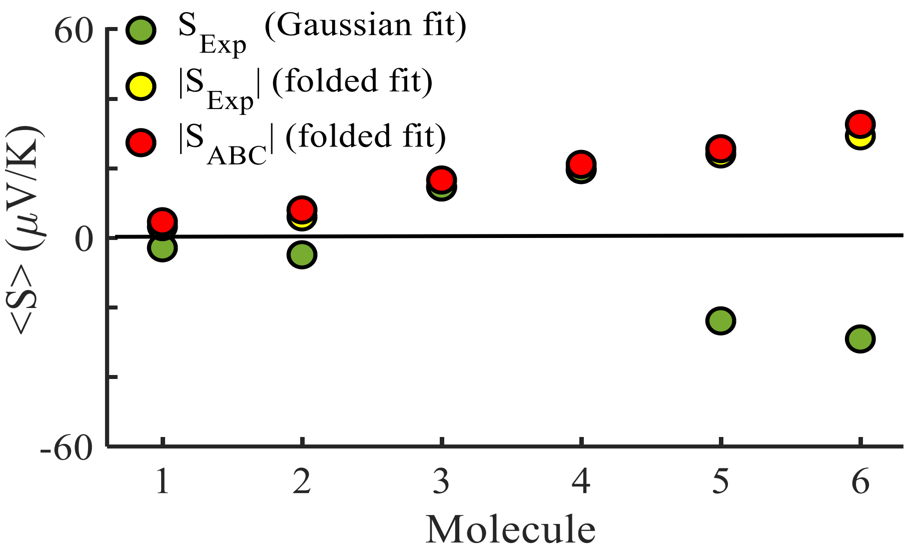

Figure S23: Experiment and theory absolute averge Seebeck coefficient <|S|> (yellow- and red-circles), measured and calculated histograms from $I - V$ curves and S experiment (green-circles)

8. The effect of the parameter c of predicted Seebeck coefficients

Herein, we compare predicted Seebeck coefficients generated when the parameter c is set to zero, with the values obtained when c is allowed to be non-zero. Table S1 shows that allowing c to be non-zero improves the agreement with experiment, though the improvement is rather slight.

Table S1: Absolute theoretical Seebeck coefficient |S| (μV/K) in two cases when c = 0 and c ≠ 0 and Absolute experiment Seebeck coefficient.

M	$\|S_{Exp}\|$	$\|S_{ABC}\|$ $(c = 0)$	$\|S_{ABC}\|$ $(c \neq 0)$
1	3.0	4.5	3.8
2	5.0	7.5	7.2
3	14.5	16.5	15.7
4	19.5	21.0	20.0
5	24.0	25.5	25.0
6	29.7	32.5	31.5

9. Seebeck coefficient obtained from $I - V$ fit versus G fit

The above results are obtained from G fit (see curve fitting section), in this section we choose 12 single $I - V$ curves (two for each molecule). Curve fitting applies directly to the $I - V$ curve to find (a, b, c) constants that are then used to calculate the Seebeck coefficient. Table S2 shows a comparison between

Seebeck coefficients obtained from different curves (G and $I-V$), the two results S_{G-fit} and $S_{I-V-fit}$ are comparable.

Table S2: Seebeck coefficient |S| (μV/K) obtained from two fits, G fit and $I-V$ fit.

**Sample** ($I-V$**curve**)	S_{G-fit}	$S_{(I-V)-fit}$	**ΔS**
**1**	2.0	2.6	0.6
**2**	1.9	2.7	0.8
**3**	3.8	4.1	0.3
**4**	2.6	3.2	0.6
**5**	2.2	2.8	0.6
**6**	2.1	2.6	0.5
**7**	7.6	8.0	0.1
**8**	1.0	1.5	0.5
**9**	3.2	3.7	0.5
**10**	3.6	4.6	1.0
**11**	3.1	3.8	0.7
**12**	2.1	2.5	0.4

10. Mean square deviations χ versus standard deviations σ

Distributions of the root mean square deviations χ_i (see Eq. 2) from each individual $G-V$ fit (i), for the 6 molecules, are shown in Figure S24. The mean values $\langle\chi\rangle$ of these values of χ_i are shown in Table S3 for each molecule. This correlation between $\langle\chi\rangle$ and Δσ is shown more clearly in Figure S25 and demonstrates that the fitting parameter $\langle\chi\rangle$ is an indicator of the accuracy of the predicted value of $|S|$ made by ABC theory.

<χ>

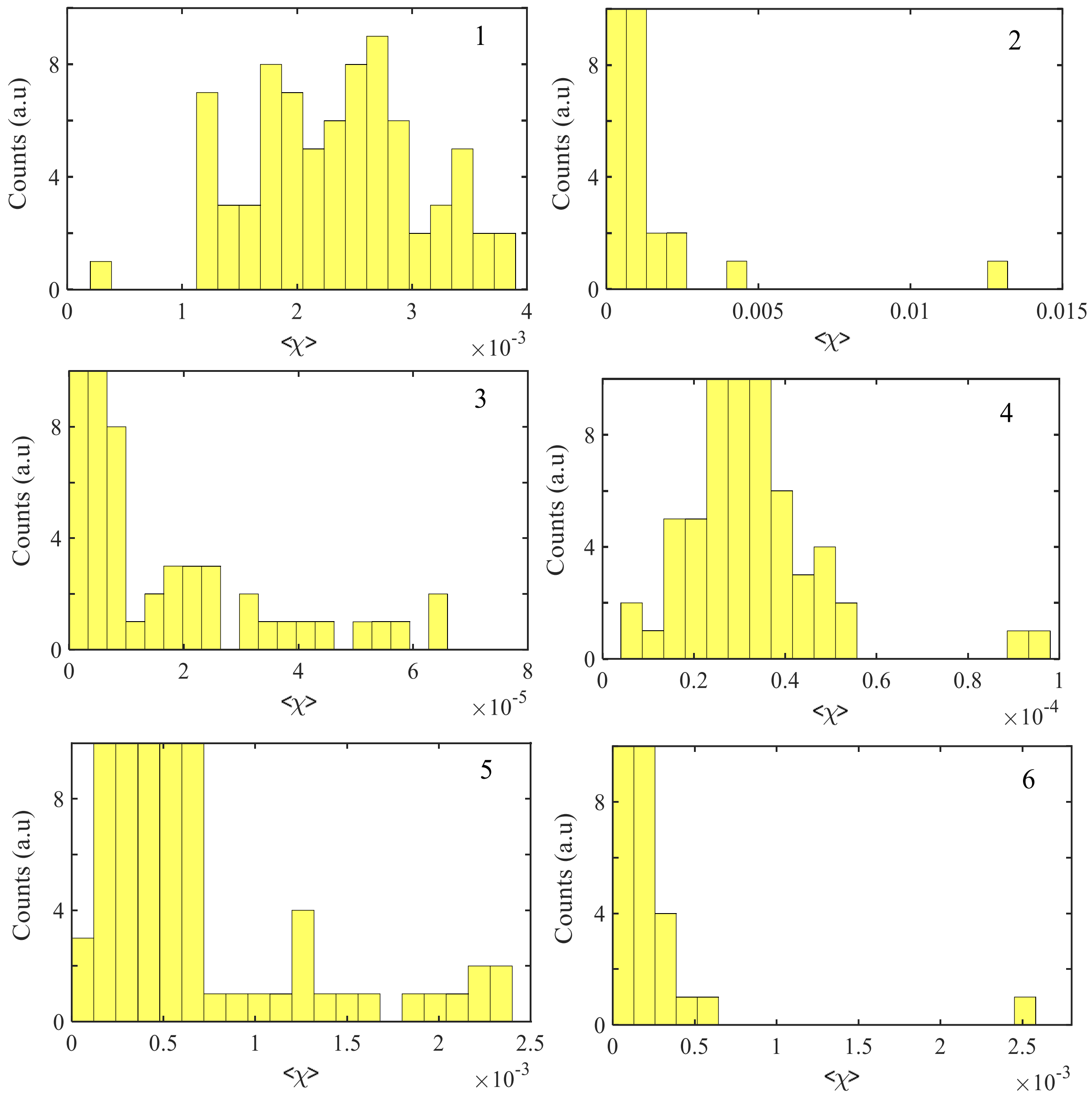

Figure S24: Distributions of root mean square deviations (χ) from individual $G-V$ fits for molecules **1-6**.

Table S3: A comparison between the differences in standard deviations between theory and experiment (Δσ), and the average root mean square deviations ($\langle\chi\rangle$) from $G-V$ fits.

M	$\Delta\sigma = \sigma_{ABC} - \sigma_{Exp}$	$\langle\chi\rangle$
1	7.71	4.0E-03
2	12.97	1.5E-02
3	0.75	6.0E-05
4	-3.26	1.0E-04
5	4.23	2.3E-03
6	7.47	2.5E-03

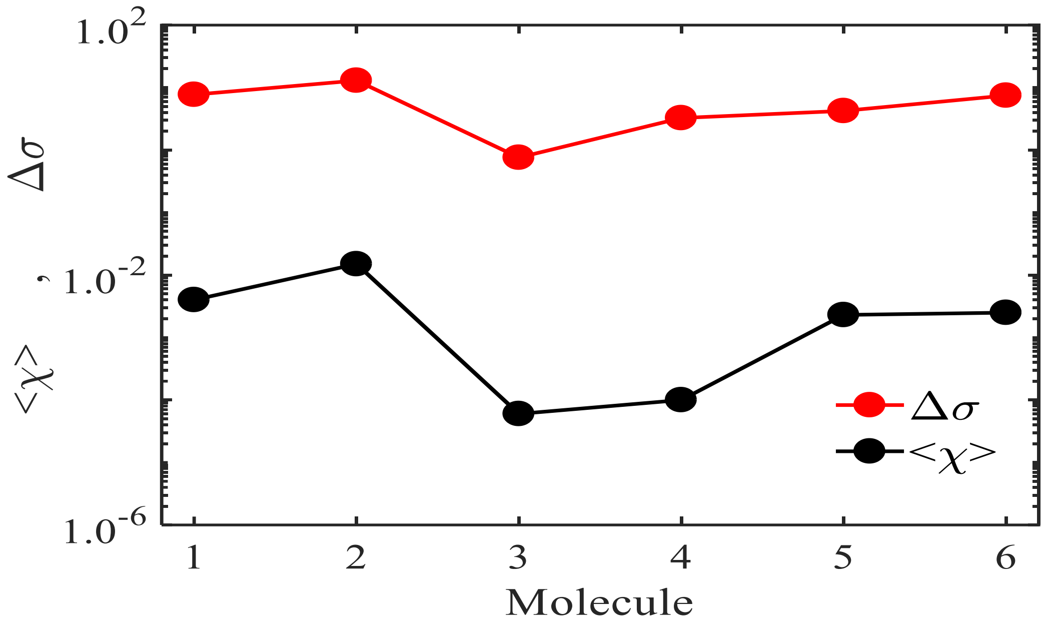

Figure S25: A plot of table 1, showing the correlation between Δσ and $\langle\chi\rangle$ for each of the 6 molecules.

Experimental measurements

11. Histograms

The experiment histogram Seebeck data $S_{Exp.}$ (green histograms of Fig.3 in the maintext), was obtained by an AFM instrument as illustrated in Figure S26 (top panel). The Seebeck histogram was obtained from a modified conductive AFM system. The substrate temperature was controlled by a Peltier stage underneath. The temperature difference between bottom substrate and top probe, ΔT, was obtained by $T_{Sample} - T_{probe}$. The yellow box in Figure S26 was the averaged thermal voltage, <V_{Therm}>, at different ΔT. Each <V_{Therm}> was averaged from ~2000 V_{Therm} data points, and the histogram in Figure s24 (bottom panel) represent the distribution of the V_{Therm} at each ΔT.

The linear fitted Seebeck Coefficient S_{linear} was obtained from the plot of <V_{Therm}> vs. ΔT, and <S> was the negative slope of the linear fit. The top panel of Figure S27 shows the measured thermal voltage at different values of ΔT. Each data point represents a measured V_{Therm} at specific ΔT (separate by colours), and the Seebeck coefficient of the corresponding point was obtained $S = -\frac{V_{Therm}(\Delta T=X)}{X}$.

A histogram of the Seebeck coefficient was obtained by collecting the Seebeck value of all data points at the bottom panel of Figure S27. The bin in the histogram with highest probability was denoted as $S_{most\ prob.}$. The histogram was fitted with Gaussian distribution, and the peak point of the Gaussian curve was denoted as $S_{Gaussian}$.

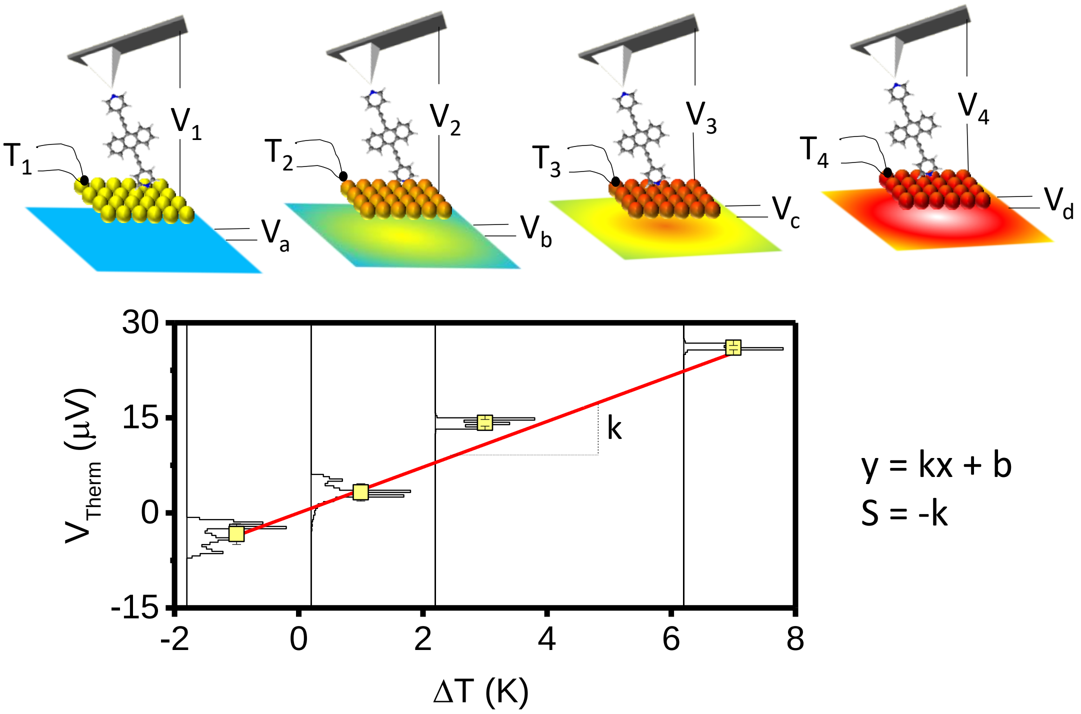

Figure S26: Scheme of instrumental setup for Seebeck coefficient measurement (**Top panel**). An example of obtaining averaged Seebeck coefficient, <S>, using linear fit method (**Bottom panel**).

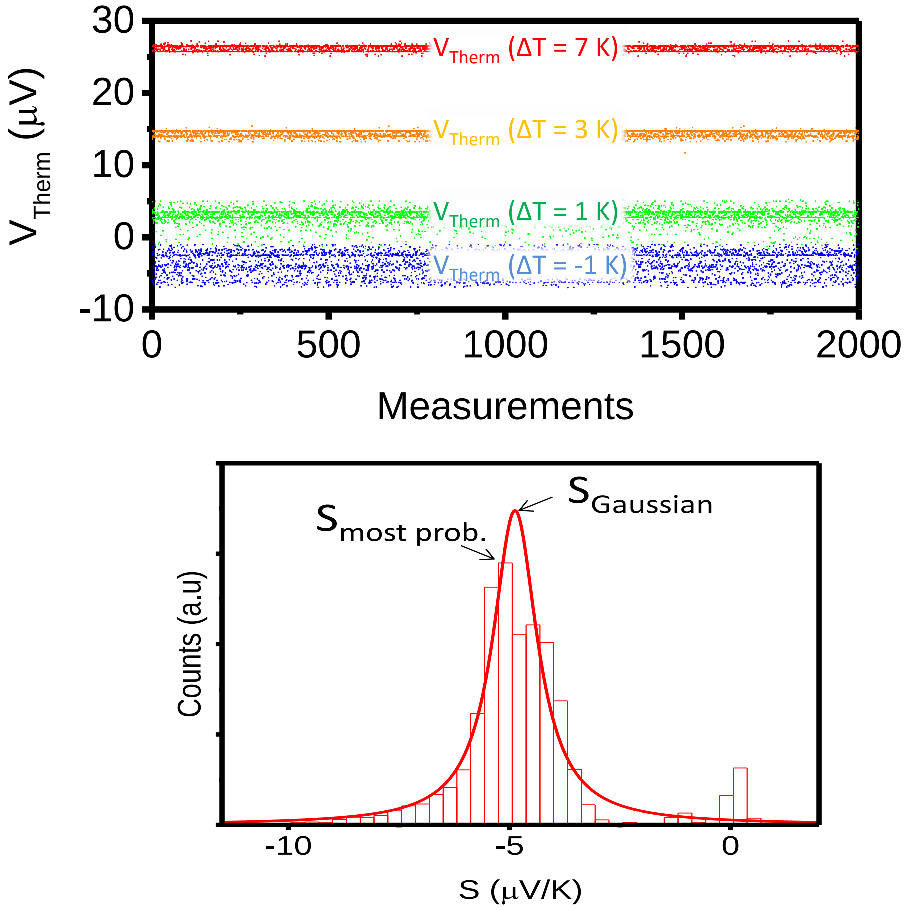

Figure S27: Collection of measured thermal voltage point at different ΔT (**Top panel**). An example of obtaining averaged the most probable value $S_{most\ prob.}$ and the Gaussian fit value $S_{Gaussian}$ (**Bottom panel**).

References

35. Wang, X.; Bennett, T. L.; Ismael, A.; Wilkinson, L. A.; Hamill, J.; White, A. J.; Grace, I. M.; Albrecht, T.; Robinson, B. J.; Long, N. J. *arXiv preprint arXiv:1911.04324* **2019**.

36. Ismael, et al. Chemical Science 2020, DOI:10.1039/D0SC02193H
